# Engineered optical and electrical performance of rf–sputtered undoped nickel oxide thin films for inverted perovskite solar cells

**DOI:** 10.1038/s41598-018-23907-0

**Published:** 2018-04-03

**Authors:** Hyeonseok Lee, Yu-Ting Huang, Mark W. Horn, Shien-Ping Feng

**Affiliations:** 1 Department of Mechanical Engineering, the University of Hong Kong, Pok Fu Lam, Hong Kong; 2 Department of Engineering Science and Mechanics, Pennsylvania State University, University Park, USA

## Abstract

Inverted perovskite solar cells incorporating RF sputtered NiO thin films as a hole transport layer and window layer are demonstrated. The electrical and optical properties of the NiO thin films are engineered using varied sputtering conditions. The localized states within bandgap owing to its crystal disorder and nonstoichiometric features affect the transmittance and the optical bandgap of the NiO thin films which in turn influences the J_sc_ of the perovskite solar cells. In addition, the electrical properties of the NiO thin films can be also varied during sputtering condition affecting the concentration of nickel vacancies and the resulting hole concentration. The conductivity largely originates from the hole concentration relating to the density of states in the NiO thin films which influence the fill factor (FF) of the solar cells. The solar cells fabricated with the NiO thin films made at 4 Pa of deposition pressure show highest performance owing to excellent transmittance and wider bandgap along with moderate conductivity. With further optimization, the perovskite solar cells exhibit ~20 mA/cm^2^ of J_sc_ and a 12.4% PCE (11.3% of averaged PCE).

## Introduction

The potential for highly0. efficient light-to-electricity conversion by organometal halide Perovskite solar cells has generated great interest for photovoltaic research since the seminal research done by Miyasaka group in 2009^1^. The progress of conversion efficiency improvement has been remarkably fast and the efficiency has already reached values comparable to that of crystalline silicon solar cells (>20%)^2,3^. The unique features of perovskite materials have facilitated this achievement: a favorable optical band gap for high efficiency (~1.5 eV), an excellent absorption coefficient in visible and near-infrared region (i.e. >10^4^ cm^−1^ at 550 nm), a low exciton binding energy of 20–30 meV, and a long carrier diffusion length of 0.1–1 µm^4–7^. For the fabrication of perovskite solar cells, n-i-p structure, conventionally, incorporating n-type TiO_2_ electron transport layer has been considered as a typical structure with p-type organic hole transport layers (HTL) such as 2,2′,7,7′-Tetrakis(N,N-di-p-methoxyphenylamine)-9,9′-spirobifluorene (spiro-OMeTAD). The n-i-p structure has contributed to the fast advance of the performance for perovskite solar cells but there are several concerns about this structure^8,9^: (1) the minuscule pinholes of the HTLs that are created after spin coating can allow foreign gas species from air to be infiltrated through the films causing an undesirable reaction with perovskite materials (2) oxygen reduction may degrade the performance of the organic HTLs themselves (3) the enhanced catalytic effect of TiO_2_ may damage the performance of perovskite materials. In addition, the n-i-p structure is vulnerable to strong hysteresis originating from several possibilities such as defect density, ferroelectricity, ion migration and/or unbalanced carrier diffusion^4,10–12^.

In this context, an inverted structure adopting different hole transport materials such as poly (3,4-ethylenedioxythiophene) poly (styrene sulfonate) (PEDOT:PSS) and nickel oxide (NiO) might provide an improved alternative. The inverted structure is formed in the structure of p-i-n and the p-i-n structure is advantageous to achieve the balanced carrier diffusion length and to reduce the hysteresis^4,10,11^. As of now, the inverted structured perovskite solar cells with PEDOT:PSS HTLs have been successfully demonstrated with high efficiency^13,14^. However, the long term stability of the perovskite solar cells is still questionable owing to the hygroscopic and acidic nature of PEDOT:PSS^4,14–16^. Hence, inorganic HTLs such as NiO would be more promising for the fabrication of efficient and long term stable perovskite solar cells owing to its inorganic natures, wide bandgap (3.6 eV), and favorable band alignment to perovskite layer^4,14,17,18^.

For the deposition of the NiO layer, solution processing through spin coating are predominant^4,14,18^. Yet, the solution process is not favorable to obtain uniform and pinhole-free films in the manner of mass production. For this reason, physical vapor deposition, such as sputtering, can be one of solutions because deposition parameters are controllable in exact manner and conformal and compact films are formed easily in the manner of mass production via this technique. Since Cui *et al*.^17^ have reported their seminal work of sputtered NiO films for the inverted perovskite solar cells, very recently, Islam *et al*.^19^ demonstrated >15% of conversion efficiency with sputtered NiO/CH_3_NH_3_PbI_3−x_Cl_x_, incorporating aluminium doped zinc oxide buffer layer. However, no sufficient understanding is provided for the specific role of the sputtered NiO thin films for the performance of solar cells and, without any doping to the perovskite, the conversion efficiency of NiO/CH_3_NH_3_PbI_3_ system is still remained lower performance than 12%. Even with doped NiO thin films made by sputtering, the performance (~12.6%) is not comparable to that of the perovskite solar cells fabricated via solution process (>16%)^18,20^.

Here we demonstrate an efficient inverted perovskite solar cell incorporating undoped NiO thin films deposited by radio frequency (RF) magnetron sputtering. The optical and electrical properties of sputtered NiO thin films are investigated as HTL and window layer. It is found that optical and electrical properties of NiO thin films are controllable by the adjustment of sputtering conditions and are affected by localized states, crystal disorder, and nonstoichiometric features. The NiO thin films prepared under 4 Pa of deposition pressure exhibit excellent optical properties and moderate electrical properties and act as efficient HTL and window layer simultaneously. Simply with thickness variation, the conversion efficiency of the inverted perovskite solar cells reached to 12.4% (11.3% of averaged PCE) owing to highly transparent and moderately conductive NiO thin films prepared by engineered deposition condition.

## Results and Discussion

**Figure 1:**
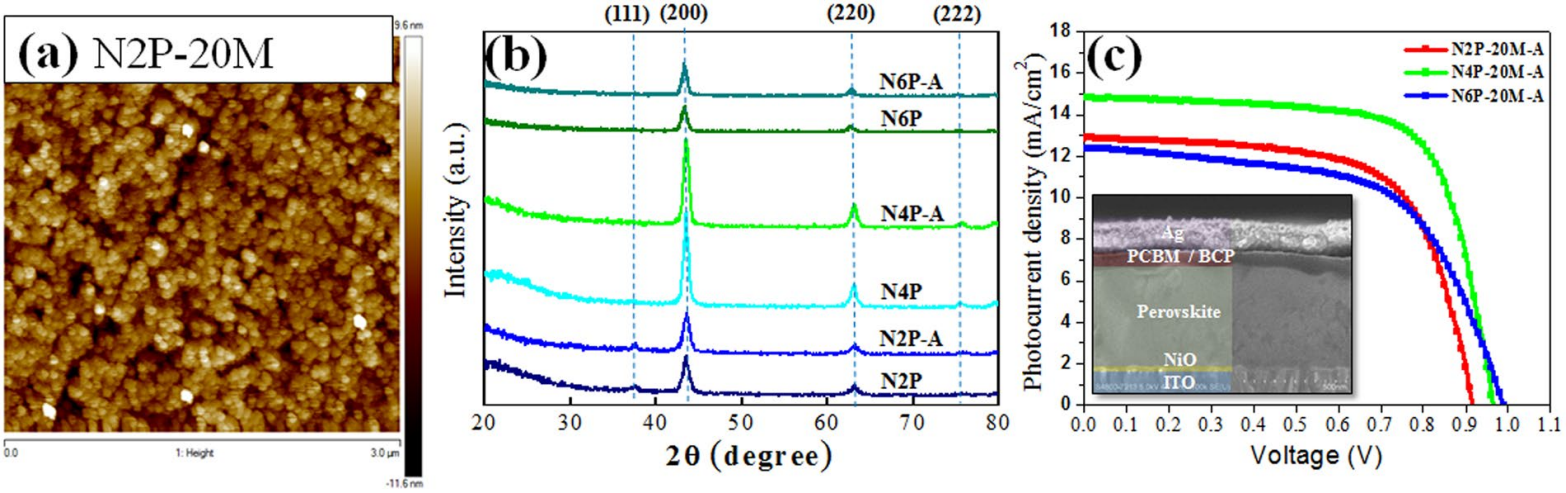
(**a**) Surface morphology of the sputtered NiO thin films for 20 min under 2 Pa of deposition pressures with annealing (**b**) X-ray diffraction pattern of 150nm-thick NiO films under varied deposition pressures. P, M, and A stands for pascal, minutes, and annealing at 200 °C for 1 h, respectively (**c**) J-V characteristics of inverted perovskite solar cells incorporating NiO thin films deposited under varied deposition pressure. The inset is the structure of the solar cells observed by field emission scanning electron microscopy.

The NiO thin films sputtered under 2, 4, and 6 Pa of deposition pressures are summarized in Table 1 with their unique names (N2P, N4P, and N6P, respectively). The NiO thin films were prepared for 20 min (20 M) at 250 W of power without or with annealing process at 200 °C for 1 h (A). Figure 1(a) shows a representative AFM image for nanoparticulated NiO which was uniformly deposited on ITO glasses with the root mean squares ranged from 3 to 4 nm for all samples (Figure S1). There is no significant difference in surface morphology as a function of deposition pressure or annealing process. X-ray diffraction (XRD) patterns were obtained with the extended deposition time to acquire 150 nm of thickness because all 20 M samples exhibited no distinguishable peak from ITO substrate due to thin thickness (≤73 nm) (Figure S2). The (111), (200), (220), and (222) growth orientations that are the features of NiO were measured from XRD pattern of 150nm-thick NiO films as shown in Fig. 1(b). Only small enhancement of intensity was observed after annealing but relatively large difference in intensity was measured in the (200) growth orientation by varied deposition pressures. The NiO thin films prepared under 4 Pa of deposition pressure (N4P and N4P-A) exhibits higher crystallinity. This indicates the deposition pressure is more influential parameter to affect the crystallinity of NiO thin films than the annealing at 200 °C for 1 h and 4 Pa of deposition pressure is more favorable in growing NiO thin films in crystallized form. It is speculated that the difference is a result of a unique features of deposition condition while sputtering. The difference in deposition rate (0.61 Å/s, 0.37 Å/s, and 0.28 Å/s for 2 Pa, 4 Pa, and 6 Pa of deposition pressure conditions, respectively) and the varied level of energetic sputtering processes that result in different forms of NiO, due to the variation in mean free path, $\ell$, of the vapor flux that can be expressed as^21^,

1 $$\ell =\frac{1}{\sqrt{2}\pi {a}^{2}}\times \frac{RT}{p{N}_{A}}$$

where $a$ is the diameter of molecule, R is the ideal gas constant, T is the absolute temperature, p is the pressure, and N_A_ is the Avogadro’s number. In other words, sputtering under lower pressure (ex. 2 Pa) is more energetic and results in a faster deposition because sputtered particles have less chance to lose their momentum or incur collisions with a longer $\ell$ while sputtering under higher pressure (ex. 6 Pa) results in more collisions and a shorter $\ell$ and is relatively less energetic with a slower deposition rate. These differences form NiO with varied crystallinity.

**Table 1:** Specification of the sputtered NiO thin films used for the investigation here. P, M, and A stand for pascal, min, and annealed at 200 °C for 1 h, respectively. N/A stands for ‘not applicable.’

Sample name	Deposition pressure (Pa)	Deposition time (min)	Annealing (°C)	Deposition rate (Å/s)
N2P-20M	2	20	N/A	0.61
N2P-20M-A	2	20	200	0.61
N4P-20M	4	20	N/A	0.37
N4P-20M-A	4	20	200	0.37
N6P-20M	6	20	N/A	0.28
N6P-20M-A	6	20	200	0.28

Photovoltaic conversion efficiency (PCE) of the perovskite solar cells incorporating the sputtered NiO thin films was evaluated under AM1.5 illumination in 0.1 cm^2^ of active area as shown in Fig. 1(c) and Table 2. The information of the measurement setup is provided in Figure S3. Here, annealed NiO thin films at 200 °C for 1 h were used for the fabrication because annealing process provides better performance^4^ and more distinguishable performance difference depending on the deposition pressure difference can be observed. The best PCE was measured from the solar cells fabricated with NiO HTLs prepared under 4 Pa of deposition pressure for 20 min (N4P-20M-A) and averaged performance was also higher than the other groups of samples. In case of the champion cell, J_sc_ is 14.83 mA/cm^2^ and PCE is 10.1%. Although both averaged and champion performances of the perovskite solar cells with the NiO thin films fabricated under 2 Pa (N2P-20M-A) and 6 Pa (N6P-20M-A) of deposition pressures were similar and those performances were poorer than that of N4P-20M-A, N2P-20M-A and N6P-20M-A showed different trend; FF of N2P-20M-A is slightly higher than N6P-20M-A while N6P-20M-A has some advantages in J_sc_ and V_oc_ on average. Since the NiO thin films applied as HTLs is the sole difference for the fabrication of solar cells, it is obvious that the NiO thin films led to these differences in the performance. The exploration on the origin of the difference from NiO thin films is essential for optimizing and enhancing the performance of solar cells further. The NiO thin films used here play an important role as HTL for the hole carriers generated from perovskite and window layer for incident photons simultaneously in the inverted structure. Hence, the NiO thin films should allow more photons to be efficiently penetrated through the films in wider range of wavelengths, which contribute more photocurrent and voltage, and generated carriers to be efficiently transferred to external circuit with less electrical loss which contribute more FF^22^.

**Table 2:** Summarization for the performance of the inverted perovskite solar cells fabricated with the NiO thin films. ave. and asterisk (*) indicate the averaged performance of the solar cells and the performance from the champion cell, respectively.

Name of the solar cells		J_sc_ (mA/cm^2^)	V_oc_ (V)	FF	PCE (%)
N2P-20M-A	ave.	10.72	0.927	0.655	6.5
		12.87	0.919	0.654	7.7*
N4P-20M-A	ave.	13.12	0.948	0.656	8.2
		14.83	0.964	0.706	10.1*
N6P-20M-A	ave.	11.89	0.959	0.540	6.2
		12.38	0.990	0.597	7.3*

**Figure 2:**
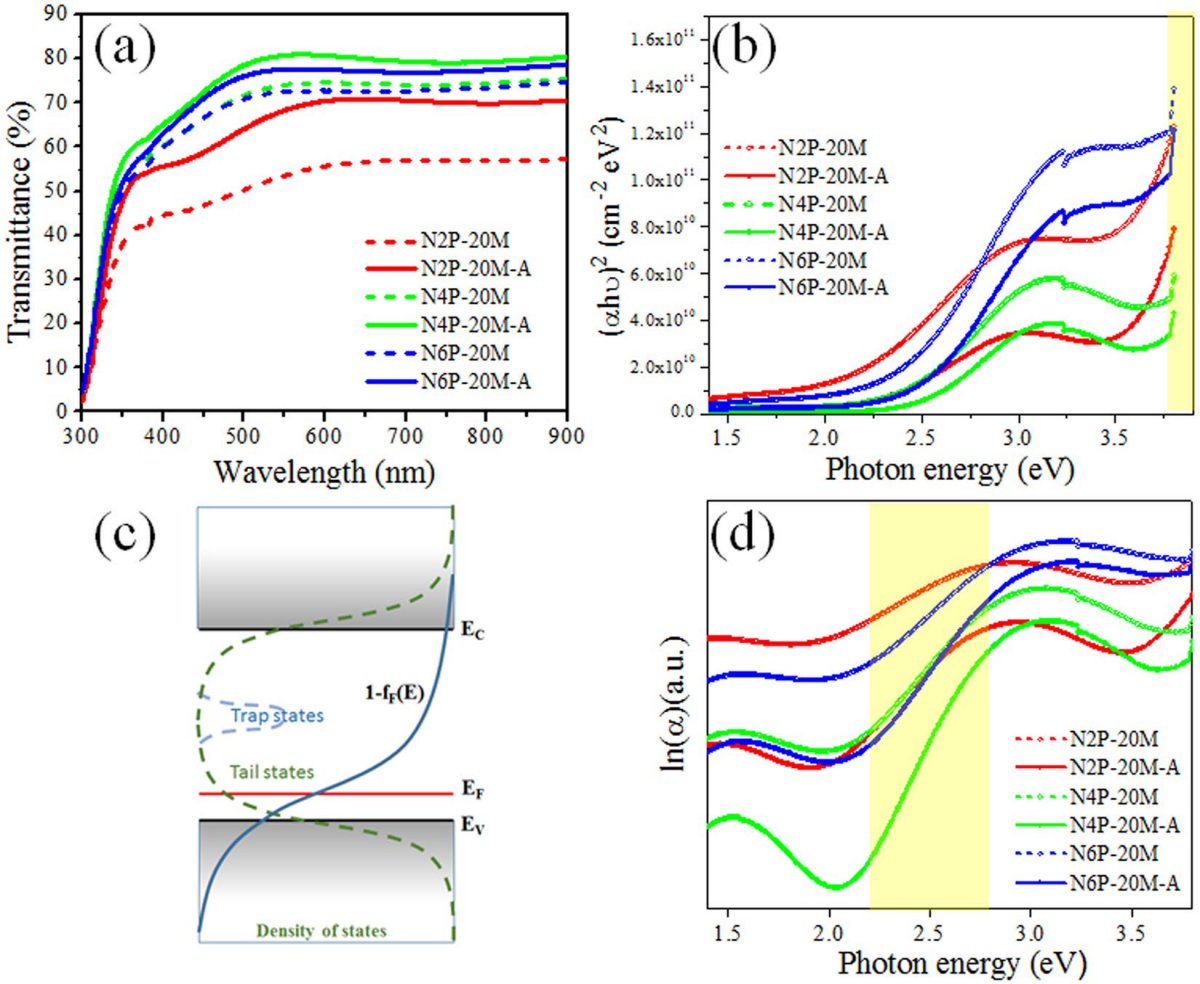
(**a**) Transmittance spectra of NiO thin films sputtered under different deposition parameters (**b**) Tauc plot of (αhν)^2^ Vs hν (**c**) the band diagram for NiO thin films (**d**) ln(α) Vs hν for NiO thin films. Yellow shaded area indicate the fitted regions for the acquisition of E_g_ and E_U_.

In this context, the NiO thin films should primarily possess higher transmittance and wider band gap. Figure 2(a) shows the transmittance spectra of the sputtered NiO thin films with the different deposition conditions as summarized in Table 1. The outstanding transmittance was observed from the NiO sample sputtered under 4 Pa of deposition pressure with annealing (N4P-20M-A) and the NiO films prepared under 2 Pa without annealing (N2P-20M) exhibited lowest transmittance through entire wavelength measured. Generally, the trend of the transmittance can be explained with the thickness of the film because the light intensity transmitted through a film is exponentially decreased by the thickness of the film, following Beer-Lambert law^22^ as in the case of the lowest transmittance of N2P-20M and N2P-20M-A (d: 73 nm). Yet, the expected highest transmittance was not measured from N6P-20M and N6P-20M-A in spite of their smallest thickness (34 nm). Rather, N4P-20M-A exhibited superior transmittance among the samples with its thicker films (44 nm). In addition to this, all samples showed enhanced transmittance only after the annealing process at 200 °C for 1 h. These indicate that other factors influenced on the result of the transmittance besides the thickness. The optical band gaps, E_g_ were obtained from absorption coefficient, and Tauc equation, considering negligible reflectance and high transparency of the ITO substrate used (see Figure S4) as shown in Fig. 2(b). The absorption coefficient, ${\rm{\alpha }}$ and Tauc equation can be expressed as follows^23,24^,

2 $${\rm{\alpha }}=-\frac{1}{d}\,\mathrm{ln}(T)$$

3 $$({\rm{\alpha }}{\rm{h}}{\rm{\upsilon }})={\rm{B}}{({\rm{h}}{\rm{\upsilon }}-{E}_{g})}^{n}$$

where d is the thickness of the films, T is the transmittance, h is the plank constant, ${\rm{\upsilon }}$ is the frequency, n is the value expressing the modes of transitions (n = 1/2 for direct bandgap here), B is a constant, E_g_ is the bandgap. The plots of (αh${\rm{\upsilon }}$)^2^ Vs h ${\rm{\upsilon }}$ were used in extracting the optical bandgaps of the NiO thin films as represented in Fig. 2(b). For as-deposited NiO thin films, the optical bandgaps were 3.41, 3.53, and 3.38 eV for N2P-20M, N4P-20M, and N6P-20M, respectively. For annealed samples, the optical bandgaps were 3.57, 3.63, and 3.49 eV for N2P-20M-A, N4P-20M-A, and N6P-20M-A, respectively. Like in the transmittance of Fig. 2(a), N4P-20M-A has a larger band gap than other samples, showing the relationship between E_g_ and crystallinity as in Fig. 1(b). More crystallized films exhibited larger E_g_. However, this could be not be explained simply by the crystallinity like in Fig. 1(b) because the annealing process also helps the increase bandgap. Another interesting finding in Fig. 2(b) is the existence of the additional linear region between 2.5 eV and 3 eV, except for the band edges as in the report from Wang *et al*.’s work^25^. The additional linear region does not correspond to the optical band gap of NiO obtained here nor reported by others^25–27^. This suggests that the existence of additional and considerable numbers of energy states within the bandgap in the form of tail states or/and trap states as described in Fig. 2(c). It is obvious that this affected the results from the transmittance and E_g_ of the NiO films in Fig. 2(a,b).

The localized states can be formed in the shape of tails over the valence band edge (E_V_) or/and below conduction band edge (E_C_) of NiO as in Fig. 2(c) due to the crystal disorder^28^. These energy states can participate in light absorption, being expressed by Urbach energy. The Urbach energy, E_U_ can be expressed as below^29,30^,

4 $${\rm{\alpha }}({\rm{E}})={\rm{Cexp}}(\frac{E}{{E}_{U}})$$

where ${\rm{\alpha }}$ is absorption coefficient and C is constant. The E_U_ of N2P-20M samples, regardless of annealing process, shows a larger number (1.37 eV and 0.88 eV for N2P-20M and N2P-20M-A, respectively) while N4P-20M has the lowest value among the samples (0.68 eV and 0.38 eV for N4P-20M and N4P-20M-A, respectively) as shown in Fig. 2(d). Namely, the N4P-20M samples possess less Urbach tails than any other samples and the annealing process helps to decrease E_U_ corresponding to their crystallinities from Fig. 1(b). In the same manner, higher temperature annealing is more beneficial to decrease the formation of the tails as shown in Figure S5. This indicates that the degree of crystal disorder is varied, relying on the deposition condition and annealing process.

**Figure 3:**
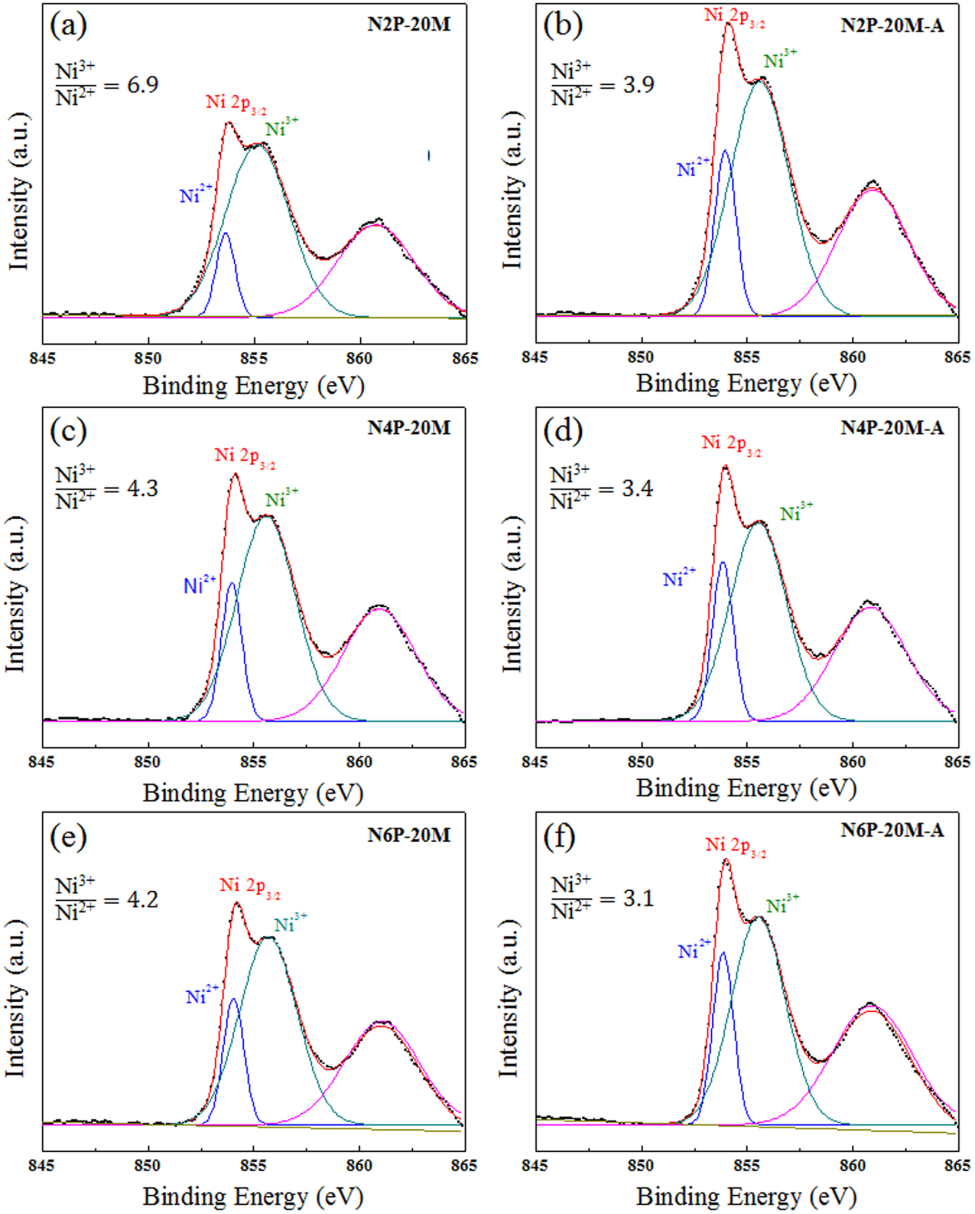
Analyzed Ni 2p_3/2_ from the sputtered NiO thin films by X-ray photoelectron spectroscopy for (**a**) N2P-20M (**b**) N4P-20M (**c**) N6P-20M (**d**) N2P-20M-A (**e**) N4P-20M-A (**f**) N6P-20M-A.

The existence of the localized states and their effect is further evidenced from the nonstoichiometric features of NiO. The hole charge carriers of NiO can be produced by the point defects like other metal oxides and especially, for Ni-poor/O-rich condition, it is highly possible that NiO can have nickel vacancy (V_Ni_) and oxygen interstitial (O_i_) as defects^31^. Yet, O_i_ may not be formed in our case because the sputtering was implemented only under Ar ambient and annealed at low temperature in ambient air. We suspect that the main source of defects is V_Ni_ for the experiment here. V_Ni_ can produce hole carriers, being ionized as acceptor-like defects (${V}_{Ni}\to {V^{\prime} }_{Ni}+{h}^{+}\,and\,{V^{\prime} }_{Ni}\to {V}_{Ni}^{^{\prime\prime} }+{h}^{+}\,where\,{V^{\prime} }_{Ni}$ is the singly ionized defect, ${V}_{Ni}^{^{\prime\prime} }$ is the doubly ionized defect, and ${h}^{+}$ is the generated hole carrier)^25^. The existence of the defects by V_Ni_ are identified by X-ray photoelectron spectroscopy (XPS) by detecting the ratio of Ni^3+^ and Ni^2+^ ions as shown in Fig. 3. The existence of Ni^3+^ suggests the existence of V_Ni_ because two Ni^2+^ ions react with oxygen and produce two Ni^3+^ ions and vacancy, following the reaction below^32–34^,

5 $$2N{i}^{2+}+\frac{1}{2}{O}_{2}\to 2N{i}^{3+}+{O}_{o}^{2-}+{V}_{Ni}$$

The binding energy of Ni^2+^ and Ni^3+^ for the Ni 2p_3/2_ peaks are located around 853.7 and 855.8 eV, respectively. An additional peak located at around 863 eV is the satellite of Ni 2p_3/2_. The obtained area ratios of Ni^3+^/Ni^2+^ were higher for the sample with lower deposition pressure and without annealing process. The ratios of Ni^3+^/Ni^2+^ were higher (>3) than reported work by others^25,35^ and these larger number suggests the existence of not only large concentration of hole carriers but also large concentration of defects from all the NiO thin films. Higher values of the ratios from N2P-20M (6.91) and N2P-20M-A (3.96) support that N2P-20M and N2P-20M-A have larger crystal disorder by huge number of the point defects. These defects can be also involved in optical absorption process, being located within the bandgap. Along with the existence of the localized states, it is evident that larger and wider distribution of localized states inside of bandgap deteriorate the optical properties of NiO thin films and higher transparency in wider range of wavelengths of NiO thin films is achievable with engineered deposition condition with post annealing process.

**Figure 4:**
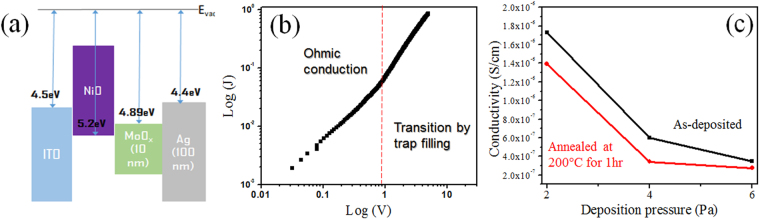
(**a**) the schematic of hole only device used here (**b**) Log(J)-Log(V) plot for NiO thin films (**c**) conductivity of the sputtered NiO thin films depending on deposition condition. The units for J and V are A/cm^2^ and V, respectively.

Another desirable property of NiO thin films for higher efficiency is the superior ability to transport the carrier generated from perovskite. Here, the electrical properties of the NiO thin films were investigated with hole only device structure to evaluate the performance as HBL. Figure 4(a) represents the schematic of the hole only device used here. All NiO thin films exhibit ohmic conduction that show the exponent, M, of 1 at lower applied voltage area with the structure and the transition of conduction mechanism owing to trap filling by carriers was observed as shown in the example in Fig. 4(b). The ohmic conduction is one of important indications that explains on efficient charge transport through NiO HTLs. The conductivity of the thin films was calculated at the ohmic conduction area with extrapolation via the equation below^36–38^,

6 $${\rm{J}}=e{\rm{\mu }}p\frac{V}{d}=\sigma \frac{V}{d}$$

where µ is the mobility of the carrier p is the hole carrier concentration, and σ is the conductivity of the films. As shown in Fig. 4(c) and Figure S6(a), obtained conductivities for the NiO thin films were 1.73 × 10^−6^ S/cm, 6.02 × 10^−7^ S/cm, and 3.48 × 10^−7^ S/cm for N2P-20M, N4P-20M, and N6P-20M, respectively. After annealing, the conductivity 1.39 × 10^−6^ S/cm, 3.43 × 10^−7^ S/cm, and 2.76 × 10^−7^ S/cm for N2P-20M-A, N4P-20M-A, and N6P-20M-A, respectively. Although the values obtained from the hole only device does not consider the effect from the interfaces and the additional resistivity from MoO_x_, the relative comparison between the samples is still meaningful. N2P-20M-A shows remarkable conductivity and that value is five times more than that of N6P-20M-A. However, no trap-filled condition was observed possibly because of relatively thin thickness (<73 nm) of the samples. Therefore, 150nm-thick NiO films under 2, 4, and 6 Pa deposition pressures were also evaluated to observe the trap-filled condition with the same hole only device as in Figure S6(b). The 150nm-thick NiO films showed trap-filled phenomena unlike the thinner NiO samples. The mobility of hole extracted from space charge limited condition (M = 2) were 2.79 × 10^−4^ cm^2^/V·s, 2.96 × 10^−4^ cm^2^/V·s, and 3.84 × 10^−4^ cm^2^/V·s for N2P-20M-A, N4P-20M-A, and N6P-20M-A, respectively. Considering the mobility is independent on the thickness variation, it is found that carrier concentration is the predominant factor that governs the conductivity of NiO thin films. The estimated carrier concentrations were 3.11 × 10^16^ cm^−3^, 7.24 × 10^15^ cm^−3^, and 4.49 × 10^15^ cm^−3^ for N2P-20M-A, N4P-20M-A, and N6P-20M-A, respectively and these values are close to theoretically calculated values for intrinsic NiO^39^. In other words, lager carrier concentration is the dominant factor for the higher conductivity of the films rather than the mobility. This finding on the carrier concentration is also consistent with the results from XPS as shown in Fig. 3. As mentioned above, because the larger concentration of Ni^3+^ led hole carrier concentration of NiO thin films, varied carrier concentrations and conductivities of the samples are also can be explained in the same manner.

**Figure 5:**
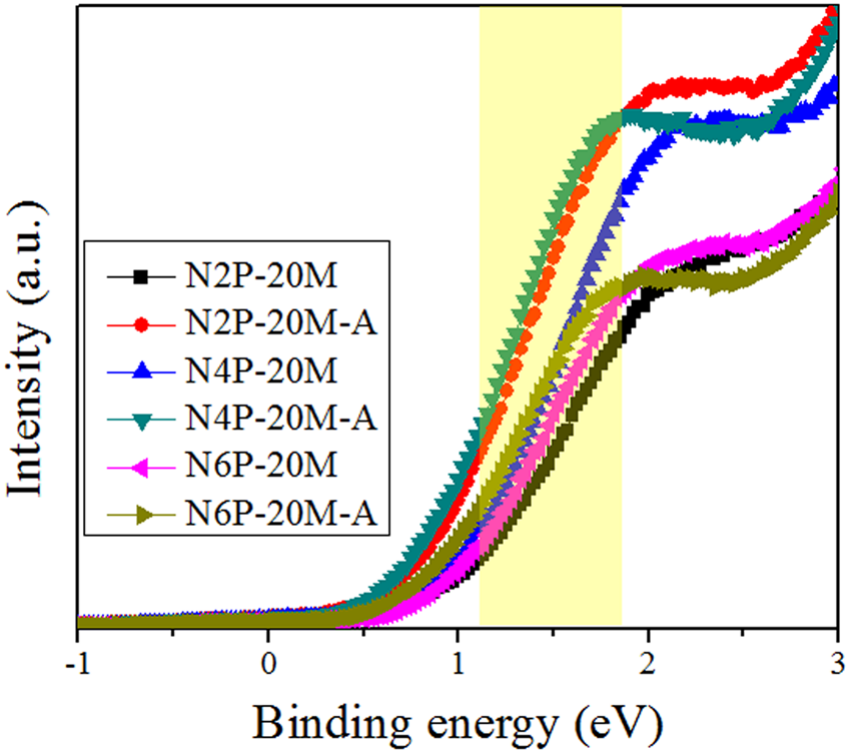
Ultraviolet photoelectron spectroscopy of NiO thin films prepared with difference conditions. Yellow shaded area indicate the fitted regions for the acquisition of E_F_ − E_V_.

Similar observations were found in the measurement by ultraviolet photoelectron spectroscopy (UPS) in Fig. 5. In general, larger hole carrier concentration is interpreted by the smaller distance between the position of Fermi level, E_F_ and that of E_V_ (namely, E_F_ − E_V_) for p-type semiconductor because Fermi-Dirac probability function, f_F_(E) is symmetric with the center of E_F_ and the probability of energy states being empty (=1 − f_F_(E)) increase as E_F_ − E_V_ decrease as in Fig. 2(c). However, the NiO thin films showed the opposite trend to this as shown in Fig. 5. As-deposited NiO films showed 0.914–0.940 eV of E_F_ − E_V_ and annealed ones showed 0.703–0.768 eV of E_F_ − E_V_. The values for E_F_ − E_V_ of NiO thin films were similar relying on annealing condition, considering the error range of extrapolation technique and, after annealing, the films show reduced values. In this situation, it is obvious that hole carrier concentration is largely affected by the density of states, following the expression below^37^,

7 $${\rm{p}}=\int g(E)\cdot [1-{f}_{F}(E)]dE$$

where $g(E)$ is the density of states in terms of energy. Different from highly crystalized materials, the density of states for NiO are affected by the localized states^40^. In the end, larger and wider distribution of localized states resulted in higher hole carrier concentration in our case. We believe that the superior electrical properties of N2P-20M and N2P-20M-A originate from larger concentration of localized states due to more energetic sputtering conditions.

From the investigation of the optical and electrical properties of NiO thin films, several essential findings have been uncovered to explain the performance difference seen in Fig. 1: (1) the performance of inverted perovskite solar cells is dependent upon the electrical and optical properties controlled by energetically sputtered NiO thin films. (2) the optical properties of NiO thin films are affected by localized states within E_g_. The existence of localized states deteriorates the transmittance because the localized states provide additional absorption. (3) the electrical properties are mainly influenced by the concentration of V_Ni_ and resulting hole concentration. Lower deposition pressure conditions provide higher conductivity owing to larger hole concentration possibly originating from larger density of states of sputtered NiO. (4) N4P-20M-A solar cells provides higher photocurrent density and enhanced performance owing to its high transmittance and moderate conductivity. (5) N2P-20M-A solar cells possess higher FF owing to higher conductivity while N6P-20M-A solar cells exhibit higher J_sc_ and higher V_oc_ due to higher transparency in spite of their lower conductivity.

**Figure 6:**
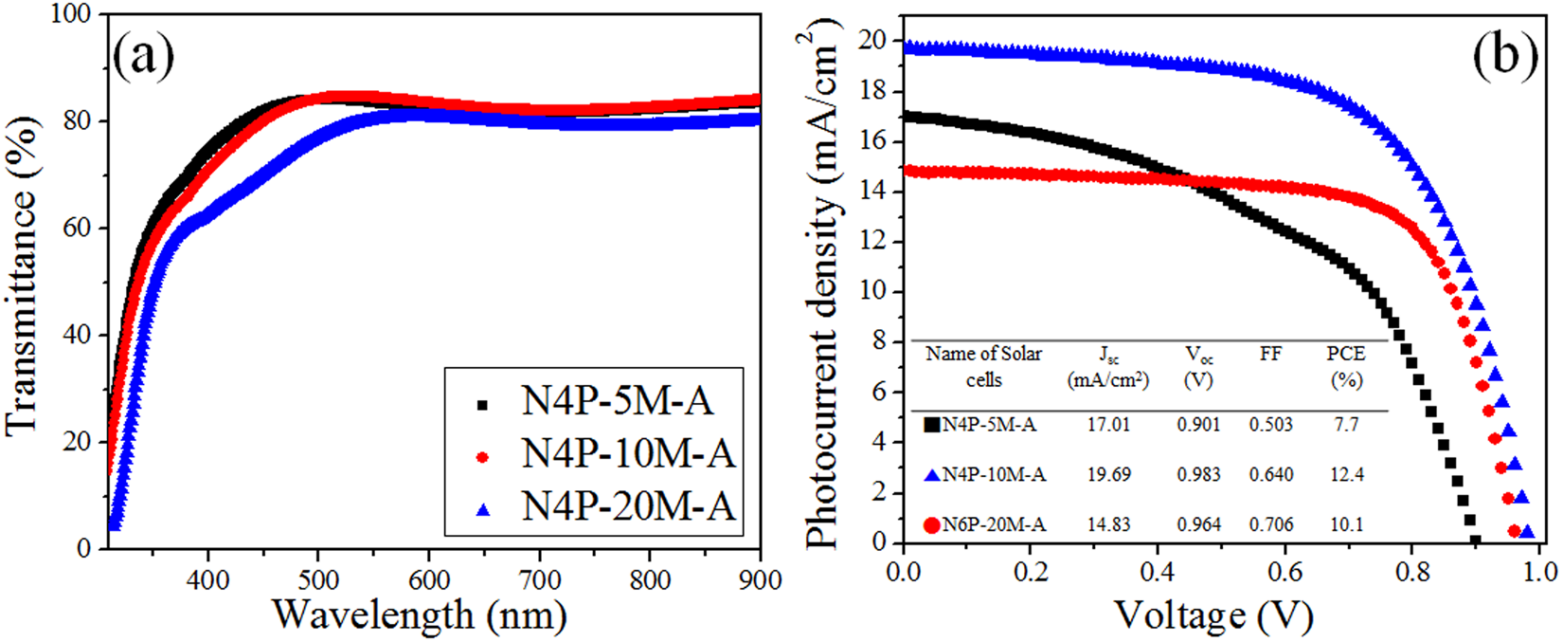
(**a**) Transmittance spectra for deposition-time varied NiO thin films (**b**) Performance of inverted perovskite solar cells depending on deposition time of NiO.

Figure 6 demonstrates the attempt for the improvement based on the findings above. The NiO thin films under 4 Pa of deposition pressure were prepared, varying deposition time to obtain more excellent optical properties with the electrical properties maintained. As shown in Fig. 6(a), the transmittance is improved as the deposition time decreases with thinner thickness and electrical properties were kept in the similar level (work function of ~4.9 eV regardless of the thickness). However, N4P-5M-A exhibits no further improvement for transmittance in the visible and near-infrared region (>450 nm). Only transmittance in shorter wavelength region is improved (<450 nm). In addition to this, in this situation, it is obvious that the series resistance is simply function of the thickness of NiO thin films. Larger thickness and smaller transmittance affect the performance degradation in N4P-20M-A. Yet, different from the expectation, N4P-10M-A solar cells exhibit more excellent PCE (12.4%) with high J_sc_ (~20 mA/cm^2^) than those from N4P-5M-A solar cells in Fig. 6(b). The averaged performance also showed the highest value (11.25%) form N4P-10M-A solar cells in Table S1, without any strong hysteresis in Figure S7(a). The reliability of these results from J-V curves is supported by the steady state performance shown in Figure S7(b). The performance of N4P-5M-A solar cells was not comparable to that of N4P-10M-A in spite of the superior transmittance and lower series resistance expected owing to its thin thickness (~11 nm). This is because the thin thickness of N4P-5M-A fails to form compact NiO films, thus producing leakage current. This is evidenced by deteriorated J_sc_ and FF of N4P-5M-A and, in a different view, this suggests that the performance of the perovskite solar cells can be further improved with more delicate control of sputter condition for NiO thin films like ultra-thin NiO thin film fabricated by atomic layer deposition^4^.

## Conclusion

Inverted perovskite solar cells incorporating RF sputtered NiO thin films without any doping or nanostructures as HTL and window layer were demonstrated. The performance of the solar cells was affected by the engineered optical and electrical properties of the NiO thin films. The properties of the NiO thin films were varied by the energetic sputtering condition associated with varied deposition pressures. It is found that the existence of localized states within bandgap, owing to its crystal disorder and nonstoichiometric feature of NiO, reduced the transmittance and the optical bandgap. This influenced the J_sc_ of the perovskite solar cells. In addition, the electrical properties of the NiO thin films can be also adjusted by the sputtering conditions. More energetic sputtering condition (ex. 2 Pa of deposition pressure) provided more conductivity to the NiO thin films because the higher concentration of nickel vacancy, V_Ni_ and resulting higher hole concentration were produced with the sputtering condition. It is found that the increased conductivity was largely originated from larger density of states of NiO thin films relating to the localized states and this contributed larger FF for the solar cells. The solar cells fabricated with the NiO thin films under 4 Pa of deposition pressure showed highest performance owing to excellent optical properties (transmittance & bandgap) and moderate conductivity. Further optimization was carried out simply with the engineered thicknesses of the NiO thin films prepared under 4 Pa of deposition pressure and the perovskite solar cells exhibited ~20 mA/cm^2^ of J_sc_ and 12.4% of PCE (11.3% of averaged PCE). We believe that this work would be a stepping stone in achieving higher efficiency for perovskite solar cells based on NiO thin films by sputtering and further improvement is possible by varying other deposition or annealing conditions.

## Methods

### RF sputtering for NiO thin films

The NiO thin films were deposited from an 80mm-diameter NiO target by RF magnetron sputter. The vacuum chamber was pumped out to <5 × 10^−4^ Pa of the base pressure and the deposition was implemented under argon atmosphere with varied deposition pressures of 2 Pa, 4 Pa, and 6 Pa. The deposition power for sputtering was 250 W and the substrates used here were indium tin oxide (ITO) glass. There is no intentional substrate heating during deposition. The NiO thin films were annealed at 200 °C for 1 h after deposition.

### Formation of Perovskite layer

The solution for the formation of the perovskite layer was prepared following ref.^18^ 2.3 g of PbI_2_, 0.8 g of CH_3_NH_3_I were mixed with 1.5 mL of DMSO and 3.5 mL of DMF and the prepared solution was stirred at 70 °C for 12 h. The solution was spread out by spin-coating at 1000 rpm for 5 s and 5000 rpm for 30 s sequentially. During the second spin-coating, 130 µl of chlorobenzene was dropped onto the samples. Subsequent annealing was carried out at 100 °C for 30 min.

### Fabrication of perovskite solar cells

The [6,6]-phenyl C61-butyric acid methyl ester (PCBM) solution (20 mg/mL in chlorobenzene) was deposited onto the perovskite layer at 1500 rpm for 30 s. Bathocuproine (BCP) layers were subsequently spin-coated at 4000 rpm for 40 s from the BCP solution (0.5 mg/mL in anhydrous absolute ethanol). Finally, a Ag metal layer was deposited by thermal evaporation.

## Electronic supplementary material

Supporting information
