# Supplementary material for: Engineered optical and electrical performance of rf–sputtered undoped nickel oxide thin films for inverted perovskite solar cells
Source: Sci Rep. 2018 Apr 3;8:5590. doi: 10.1038/s41598-018-23907-0 (PMC5882863; doi:10.1038/s41598-018-23907-0)
Supplement: Supplementary file 1 — Supporting information [file 41598_2018_23907_MOESM1_ESM.pdf]

Supporting information

Engineered optical and electrical performance of rf–sputtered undoped nickel oxide thin films for inverted perovskite solar cells

Hyeonseok Lee[a1], Yu-Ting Huang[a1], Mark W. Horn[b], Shien-Ping Feng[a*]

[a]Department of Mechanical Engineering, the University of Hong Kong, Hong Kong

[b]Department of Engineering Science and Mechanics, the Pennsylvania State University, University Park, USA

*Corresponding author

Email address: hpfeng@hku.hk (S.-P. Feng)

[1]These authors have contributed equally

Characterization

Field emission scanning electron microscopy (LEO 1530) was used to investigate the surface morphologies of sputtered NiO thin films. Grazing incidence X-ray diffraction (Rigaku, smartlab) was conducted to identify the crystal structures of NiO thin films. UV-vis spectroscopy (Perkin Elmer, Lambda 35) was used to measure the optical properties of the samples including transmittance, optical bandgap, and

Urbach energy. Measurement of photovoltaic performance was performed using a Keithley 2400 source meter under AM 1.5 illumination (100 mW/cm^2, Peccell); specifically, the short circuit current, open circuit voltage, fill factor, and conversion efficiency of the perovskite solar cells were measured. X-ray photoelectron spectroscopy (XPS) is used to determine Ni^{3+}/Ni^{2+} ratio. UV photoelectron spectroscopy (UPS) was implemented to determine the position of the work function and valence band. A bias of –10 V was applied to the samples, and 21.22 eV of He (I) line was used. An SKP5050 scanning Kelvin probe system (KP Technology Ltd.) with a resolution of 1–3 meV was used for the work function measurement. Highly ordered pyrolytic graphite was used as a reference (4.6 eV) to calculate the absolute work function values of different carrier transport layers.

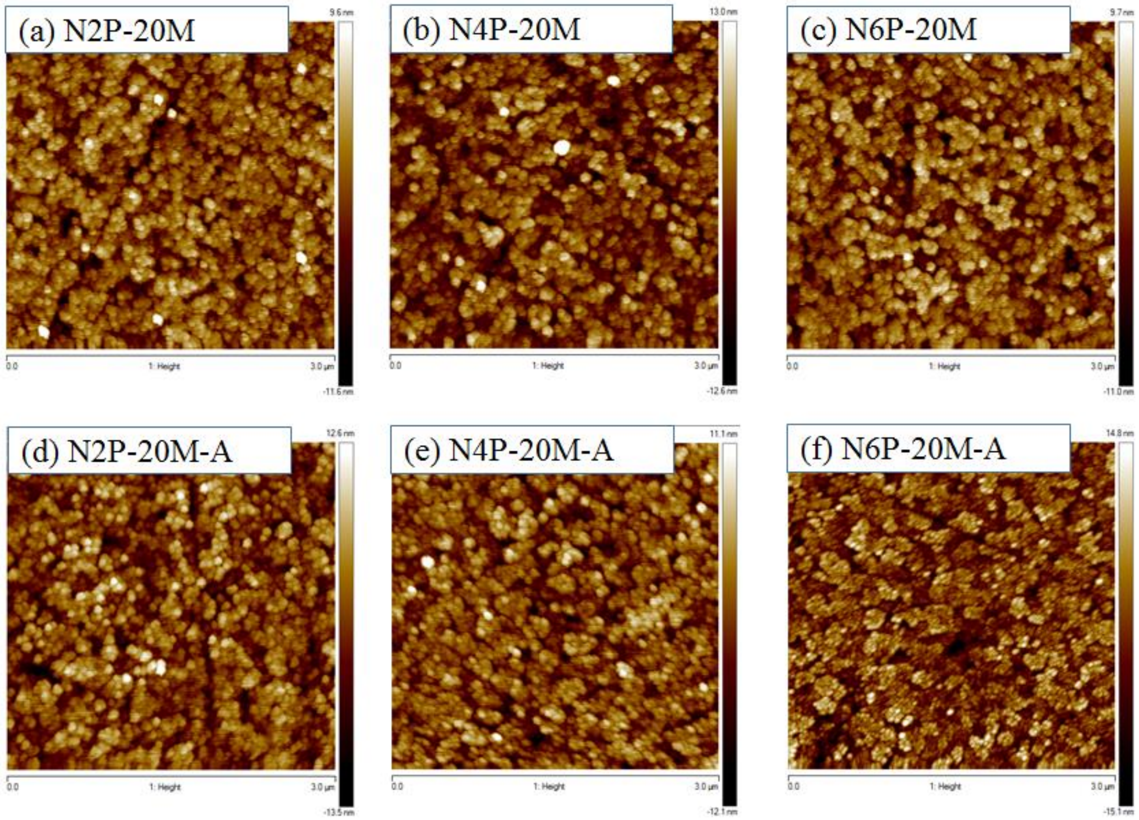

Figure S1. Surface morphologies of the sputtered NiO thin films prepared under varied deposition pressure with or without annealing process by atomic force microscopy.

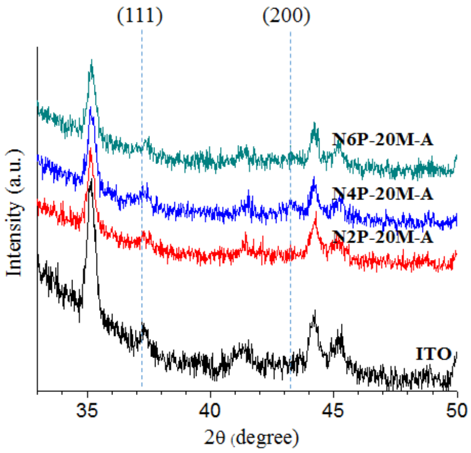

Figure S2. X-ray diffraction pattern for the NiO films sputtered for 20 min under varied deposition pressures. P, M, and A stands for pascal, min, and annealing at 200 °C for 1h, respectively.

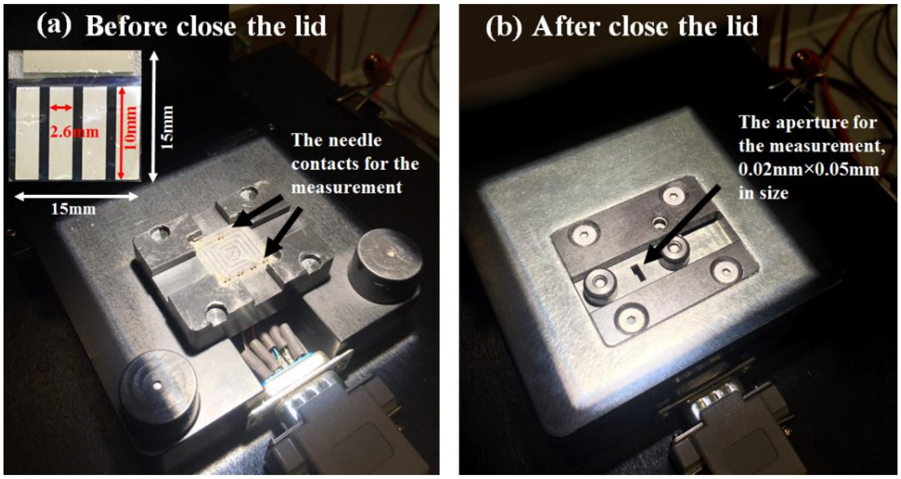

Figure S3. The measurement setup for perovskite solar cells (a) before close the lid and (b) after close the lid. The inset in Figures S1 shows the size of the solar cell and metal contact we used here. Four single cells are measured separately.

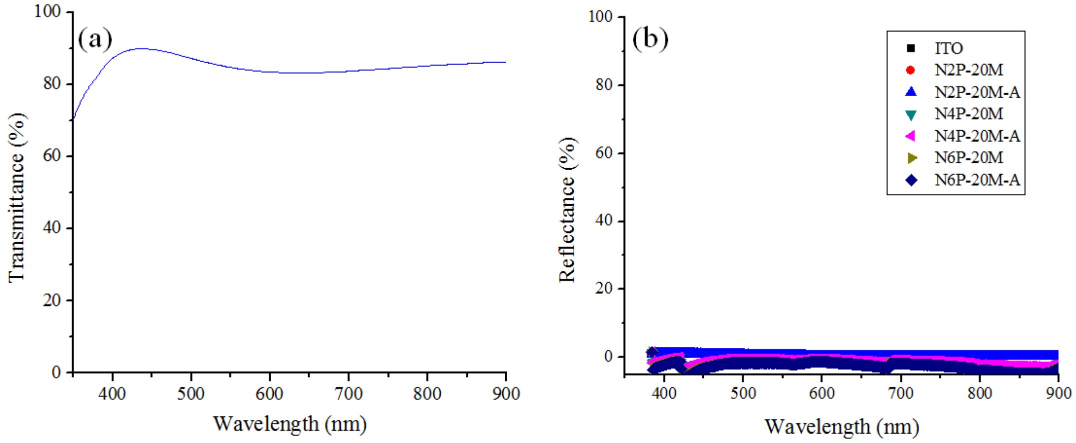

Figure S4. (a) Transmittance spectra of ITO substrate and (b) reflectance spectra for the sputtered NiO thin films.

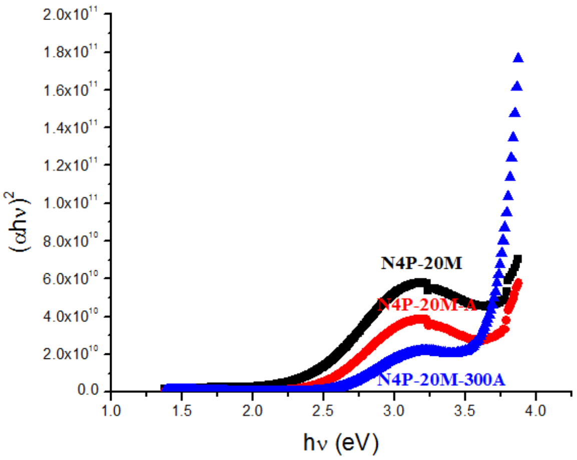

Figure S5. Tauc plots of $(\alpha h\nu)^2$ vs hν for N4-20M, N4-20M-A, and N4-20M-300A. 300A indicates the NiO thin films annealed at 300 °C for 1h.

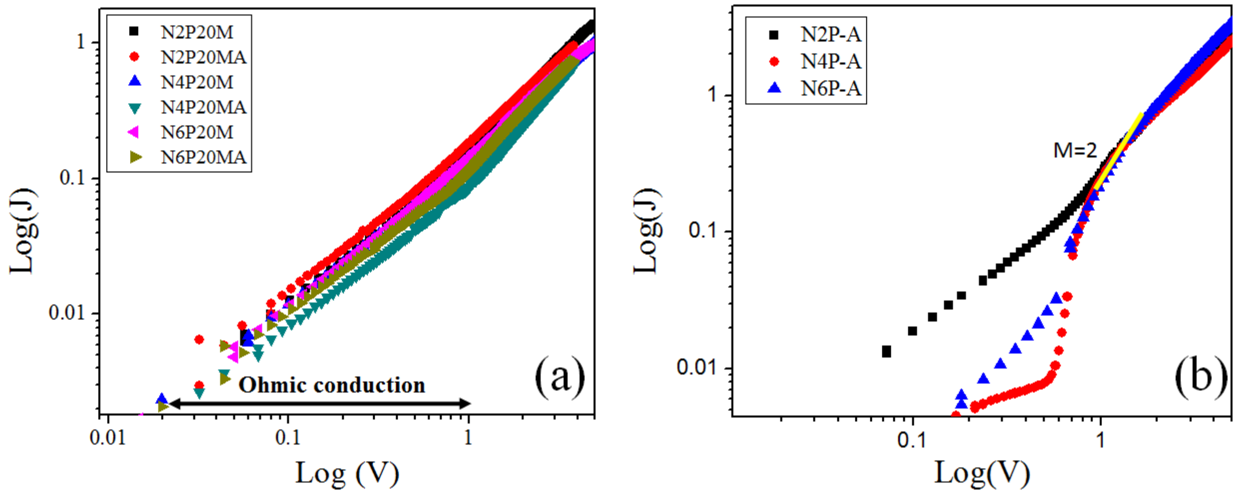

Figure S6. Plots for Log (J) Vs Log(V) from (a) the NiO thin films deposited for 20 min and (b)150nm-thick NiO films with varied deposition pressures.

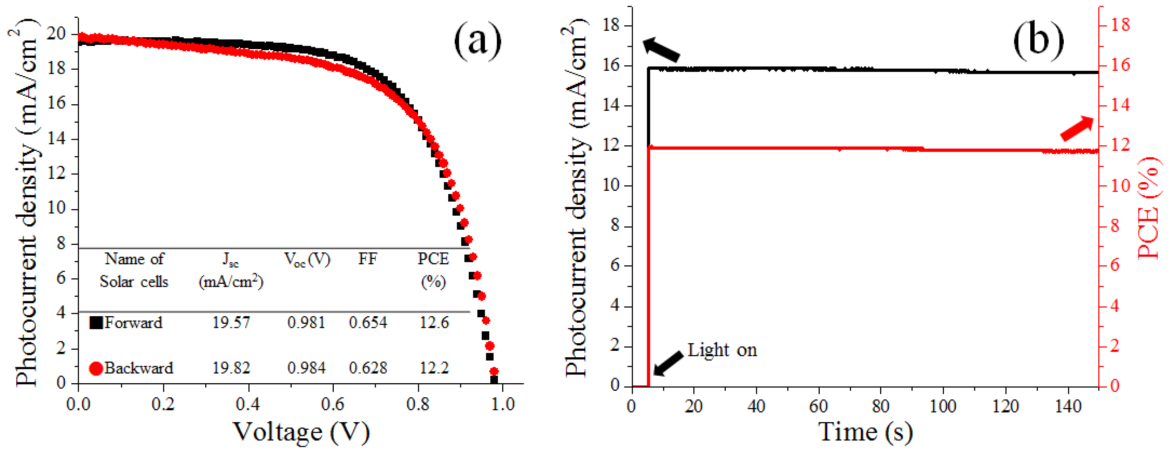

Figure S7. (a) Forward (J_{sc}→V_{oc}) and Backward (V_{oc}→J_{sc}) scan of PCE and (b) Steady state Photocurrent density (red) and PCE for N4P for N4P-10M-A with 0.75V of the applied bias.

Name of the solar cells		J_{sc} (mA/cm^2)	V_{oc} (V)	FF	PCE (%)
N4P-5M-A	ave.	15.36	0.878	0.467	6.3
		17.01	0.901	0.503	7.7*
N4P-10M-A	ave.	18.31	0.965	0.636	11.3
		19.69	0.983	0.640	12.4*
N4P-20M-A	ave.	13.12	0.948	0.656	8.2
		14.83	0.964	0.706	10.1*

Table S1. Summarization for the performance of the inverted perovskite solar cells with the NiO thin films sputtered for different deposition time. Asterisk(*) and ave. indicate the performance from the champion cell and the averaged performance from more than 10 cells, respectively.
